# Metagenomic insights and biosynthetic potential of *Candidatus* Entotheonella symbiont associated with *Halichondria* marine sponges

**DOI:** 10.1128/spectrum.02355-24

**Published:** 2024-11-22

**Authors:** Hiyoung Kim, Jiyeong Ahn, Jaebum Kim, Hahk-Soo Kang

**Affiliations:** 1 Department of Biomedical Science and Engineering, Konkuk University, Seoul, South Korea; University of Porto, Porto, Portugal

**Keywords:** marine sponges, symbionts, metagenomics, biosynthetic gene clusters

## Abstract

Korea, being surrounded by the sea, provides a rich habitat for marine sponges, which have been a prolific source of bioactive natural products. Although a diverse array of structurally novel natural products has been isolated from Korean marine sponges, their biosynthetic origins remain largely unknown. To explore the biosynthetic potential of Korean marine sponges, we conducted metagenomic analyses of sponges inhabiting the East Sea of Korea. This analysis revealed a symbiotic association of *Candidatus* Entotheonella bacteria with Halichondria sponges. Here, we report a new chemically rich Entotheonella variant, which we named *Ca*. Entotheonella halido. Remarkably, this symbiont makes up 69% of the microbial community in the sponge *Halichondira dokdoensis*. Genome-resolved metagenomics enabled us to obtain a high-quality *Ca*. E. halido genome, which represents the largest (12 Mb) and highest quality among previously reported *Entotheonella* genomes. We also identified the biosynthetic gene cluster (BGC) of the known sponge-derived Halicylindramides from the *Ca*. E. halido genome, enabling us to determine their biosynthetic origin. This new symbiotic association expands the host diversity and biosynthetic potential of metabolically talented bacterial genus *Ca*. Entotheonella symbionts.

**IMPORTANCE:**

Our study reports the discovery of a new bacterial symbiont *Ca*. Entotheonella halido associated with the Korean marine sponge *Halichondria dokdoensis*. Using genome-resolved metagenomics, we recovered a high-quality *Ca*. E. halido MAG (Metagenome-Assembled Genome), which represents the largest and most complete *Ca*. Entotheonella MAG reported to date. Pangenome and BGC network analyses revealed a remarkably high BGC diversity within the *Ca*. Entotheonella pangenome, with almost no overlapping BGCs between different MAGs. The cryptic and genetically unique BGCs present in the *Ca*. Entotheonella pangenome represents a promising source of new bioactive natural products.

## INTRODUCTION

**Fig 1:**
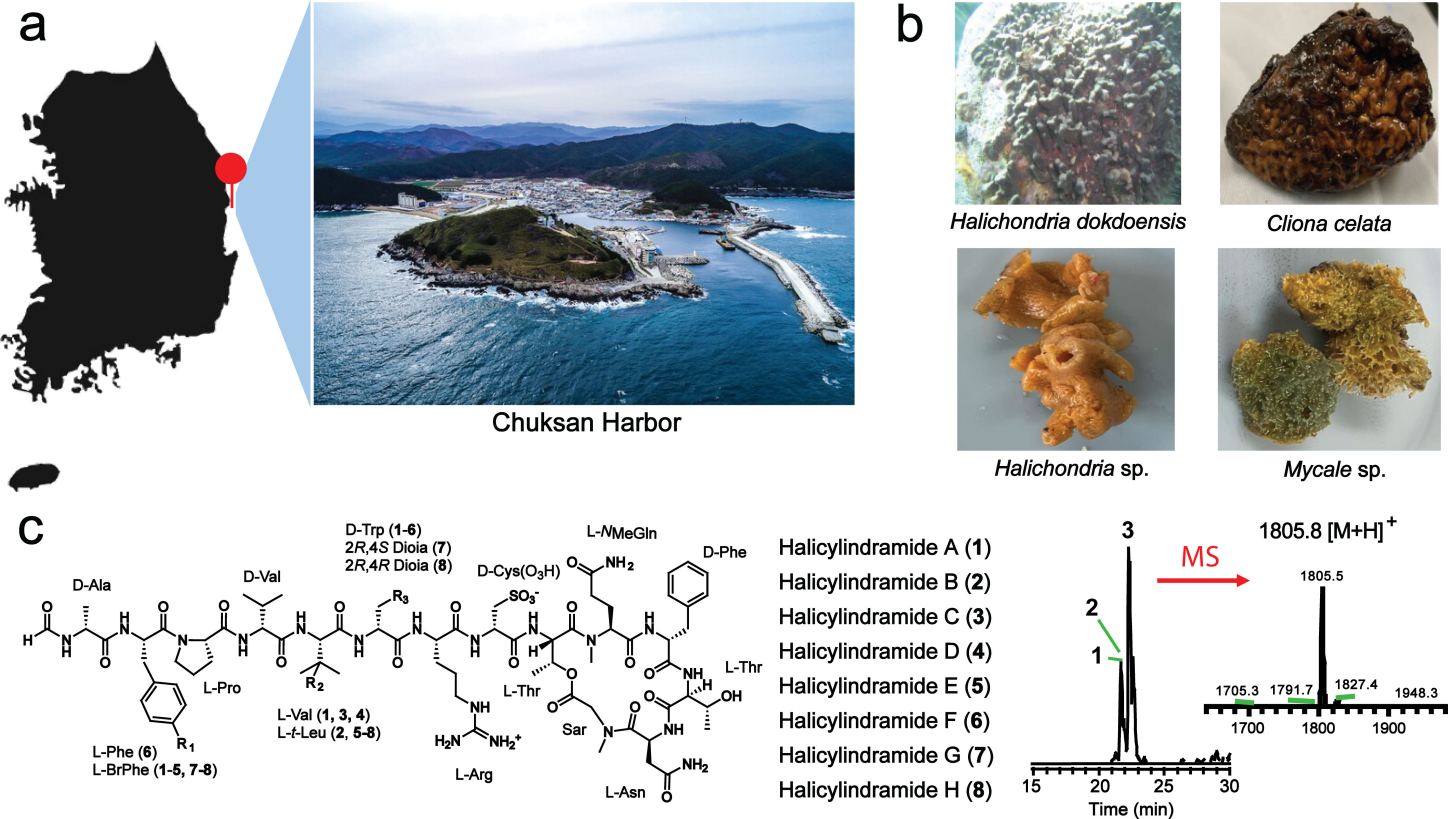
Halicylindramides from the marine sponge *Halichondria dokdoensis*. (a) The collection site is located in the East Sea of Korea, marked by a red pin on the map. This specific area is home to diverse native Korean marine sponge species. To minimize environmental and seasonal biases, all sponge samples were collected from the same location simultaneously. (b) Photographs of the collected marine sponges. These sponges were deposited at the National Marine Biodiversity Institute of Korea. The taxonomy was determined by a combination of molecular data and morphological traits. (c) Chemical structures of the halicylindramides. Of the four collected sponges, *Halichondria dokdoensis* was previously known to be the producer of the peptide natural products, halicylindramides. The presence of halicylindramides in *H. dokdoensis* was confirmed by LC-MS analysis of the organic extract. Halicylindarmide C was the major congener.

Marine sponges are ancient organisms that host a rich diversity of microbial life, contributing significantly to the ecological dynamics of marine ecosystems. These sponges have been known to be a prolific source of bioactive natural products with origins often linked to their microbial symbionts (1, 2). However, the difficulty of culturing these symbionts in laboratories makes it challenging to assess their chemistry. Metagenomic exploration offers a viable alternative to reveal their true biosynthetic potential by analyzing biosynthetic gene clusters (BGCs) found in sponge metagenomes (3). The East Sea of Korea is a unique marine environment characterized by a complex coastal geography where the warm Kuroshio current converges with the cold North Korean current (4). These unique geographical conditions create a rich habitat for marine sponges (Fig. 1a). According to statistical data from the Korean National Institute of Biological Resources, a total of 432 sponge species from the phylum Porifera alone have been identified (5). These Korean native marine sponges have been sources of many bioactive natural products, such as haliclonin, monanchosterols, and gombamide A (6–13). Despite the isolation of a diverse array of bioactive compounds, their biosynthetic origins remain largely unknown. Previous studies have shown that sponge-associated microbial symbionts are the true sources of many sponge-derived natural products (14–16). Therefore, metagenomic exploration of Korean marine sponges could reveal the diversity as well as biosynthetic potential of their associated microbial symbionts.

*Halichondria dokdoensis,* initially classified as *Petrosia* sp., has been recently identified as a new sponge species living in Korea (17). This sponge species, found in high abundance in the coastal area of the East Sea, has previously been identified as the producer of the cyclic depsipeptide natural products named halicylindramides (7), which also have been isolated from the closely related Japanese marine sponge *Halichondria cylindrata* (18, 19). Eight halicylindramides have been reported to date with a wide range of biological activities, including antifungal activity against *Mortierella ramanniana*, cytotoxicity against murine leukemia cells, and antagonistic activity against human farnesoid X receptor (hFXR) (7, 18, 19). These diverse biological activities suggest that halicylindramides may play a defensive role for their hosts, but their actual producer and biosynthetic origin have been unknown, hampering further studies. The presence of non-standard D- and *N*Me-amino acids suggests that these natural products are likely to be synthesized by a non-ribosomal peptide synthetase (NRPS). As NRPSs are exclusively found in bacteria, the actual producer of halicylindramides is likely to be a bacterial symbiont residing within the sponge. The identification of their bacterial source and BGCs would therefore offer an opportunity for the detailed study of biological activities by enabling heterologous production and structural derivatization.

In this study, we investigated the microbial community of four Korean marine sponges, all collected from the same location in the East Sea of Korea. A 16S rDNA-based taxonomic analysis of the sponge microbiomes revealed that each sponge harbors a distinct microbial community, despite being collected from a geographically close coastal area. Notably, we found that the Korean *Halichondria* sponges exclusively harbor bacterial symbionts of the genus “*Candidatus* Entotheonella.” To gain insight into the phylogenetic position and biosynthetic potential of these *Ca*. Entotheonella symbionts, we sequenced the metagenome of *H. dokdoensis*. The hybrid assembly of short-read (Illumina) and long-read (PacBio) sequencing data led to the recovery of five high-quality metagenome-assembled genomes (MAGs). The bioinformatics analysis of *Ca*. Entotheonella MAG identified the NRPS BGC with a module organization corresponding to the amino acid composition of the halicylindramides. Then, we successfully cloned this BGC into a bacterial artificial chromosome (BAC) vector, setting a foundation for future synthetic biology efforts to express this BGC in a culturable, genetically tractable heterologous host.

## RESULTS AND DISCUSSION

### Microbial community of native Korean marine sponges

**Fig 2:**
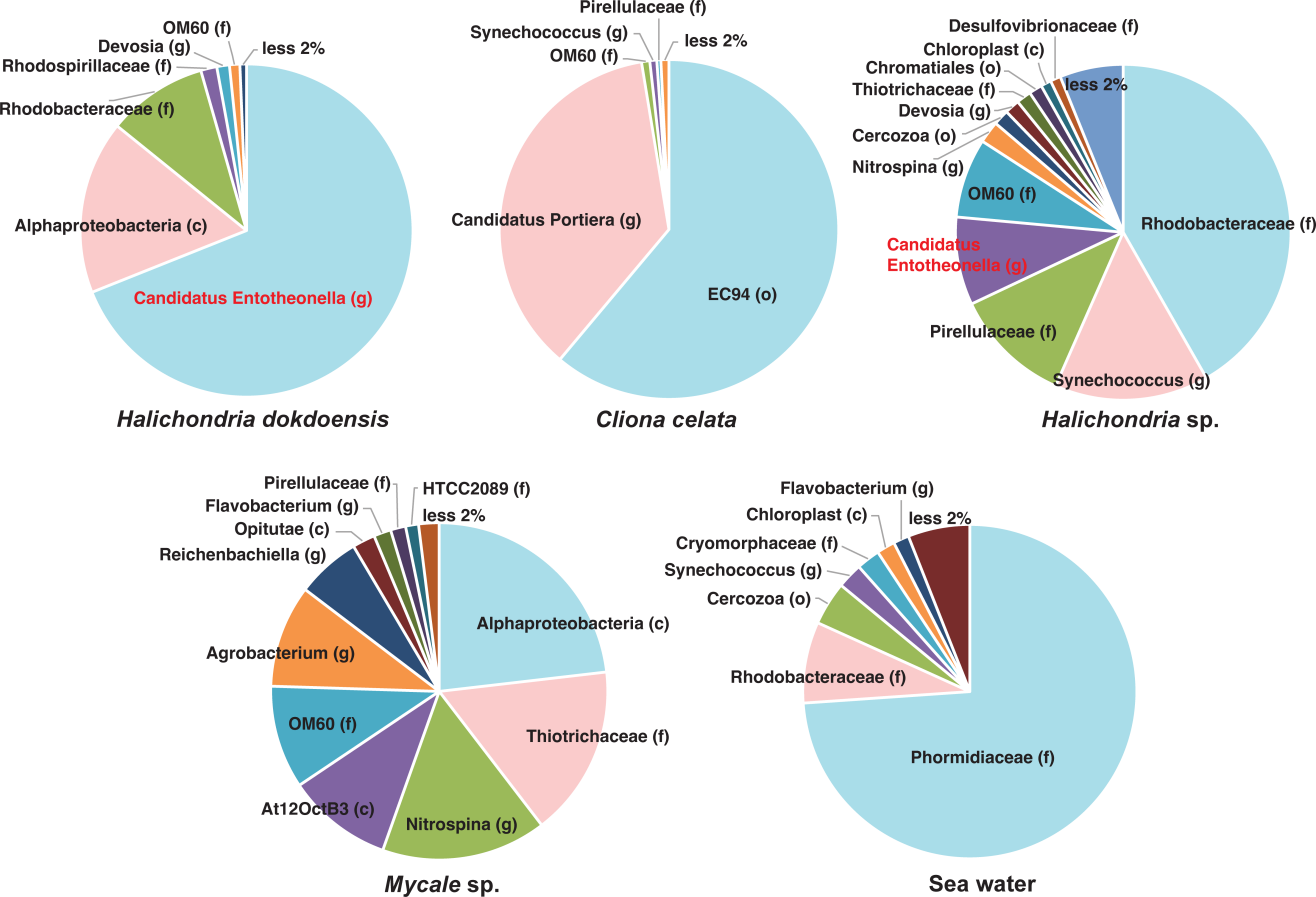
Microbial community analysis of four Korean native marine sponges. The microbial community composition of the four native Korean marine sponges (*Halichondria dokdoensis*, *Cliona celata*, *Halichondria* sp., and *Mycale* sp.) as well as the surrounding seawater was analyzed using full-length 16S rRNA gene amplicon sequencing on the PacBio platform. The pie charts illustrate the composition at the lowest identified taxonomic levels: class (c), order (o), family (f), genus (g), and species (s). Pie charts were generated to depict the relative abundance of each taxon, with colors assigned according to their rank in abundance. Notably, the genus *Ca.* Entotheonella comprises approximately 69% of the total microbial community in the metagenome of *H. dokdoensis*.

To gain insight into the diversity of sponge-associated microbiomes, we first performed the microbial community analysis of four native marine sponges, all collected from the same coastal area of the East Sea (Fig. 1a and b). The presence of halicyclindramides A-C (1–3) in *H. dokdoensis* was confirmed by LC-MS analysis of the organic extract (Fig. 1c). Then, full-length 16S rDNA sequences were PCR-amplified from the metagenomic DNA samples using universal primers and subsequently sequenced on the PacBio Sequel platform. This yielded HiFi read data in the range of 51.3–81.6 Mb per sample, with average read lengths of 1.45–1.50 kb. An overall sequencing quality based on a Phred score of Q30 indicated a base call accuracy of 99.9% (1 error in 1,000 bases) (Table S1). These sequencing reads were processed and clustered into operational taxonomic units (OTU) at the species level (97% identity) using the SILVA SSU reference library (20). The taxonomic analysis of sequencing data showed distinct microbial community compositions among different sponges, with most members not detected in the surrounding seawater (Fig. 2; Fig. S1). The most abundant member in the seawater was the family Phormidiaceae belonging to the phylum Cyanobacteria, which was not present in any of the four sponge microbiomes. The family OM60, belonging to the phylum Proteobacteria, was the most common sponge-associated microbial member as found in all the Korean sponges but not in the Seawater. We also identified several sponge-specific microbial members. The sponge *Clinona celata* exhibited a microbial composition composed of 97% Beta- and Gamma-proteobacteria, of which 60% belonged to the order EC94 (recently renamed to *Candidatus* Tethybacterales) (21). The order Tethybacterales is known to consist predominantly of sponge-associated symbionts, indicating significant adaptation and co-evolution with sponges. The sponge *Mycale* sp. exhibited the most diverse microbial community composition, with a high abundance of members known for nutrient cycling in marine environments, such as the family Thiotrichaceae and OM60, and the genus Nitrospina and Reichenbachiella (22–24). The most notable feature was the association of the genus *Ca*. Entotheonella with the two morphologically distinct *Halichondria* sponges, *Halichondria dokdoensis,* and *Halichondria* sp. The relative abundance of the *Ca*. Entotheonella symbiont was 8% in *Halichondria* sp, but it was unprecedently high in *H. dokdoensis*, constituting 69% of the total microbial community (Fig. 2; Fig. S1). Microscopic observation of the sliced sponge revealed that the *Ca*. Entotheonella symbiont is highly abundant in the sponge mesophyl as characterized by its large, filamentous morphology (Fig. S2) (25). Although the sponge-associated *Ca*. Entotheonella species has been reported to be the metabolically talented bacterial genus, they have been dominantly found in the sponge *Theonella swinhoei* (26–30). The symbiotic presence of *Ca*. Entotheonella species in the sponge *H. dokdoensis* implies that they have adapted to a wider range of marine sponge hosts.

### Genome-resolved metagenomics of *H. dokdoensis* sponge

**Fig 3:**
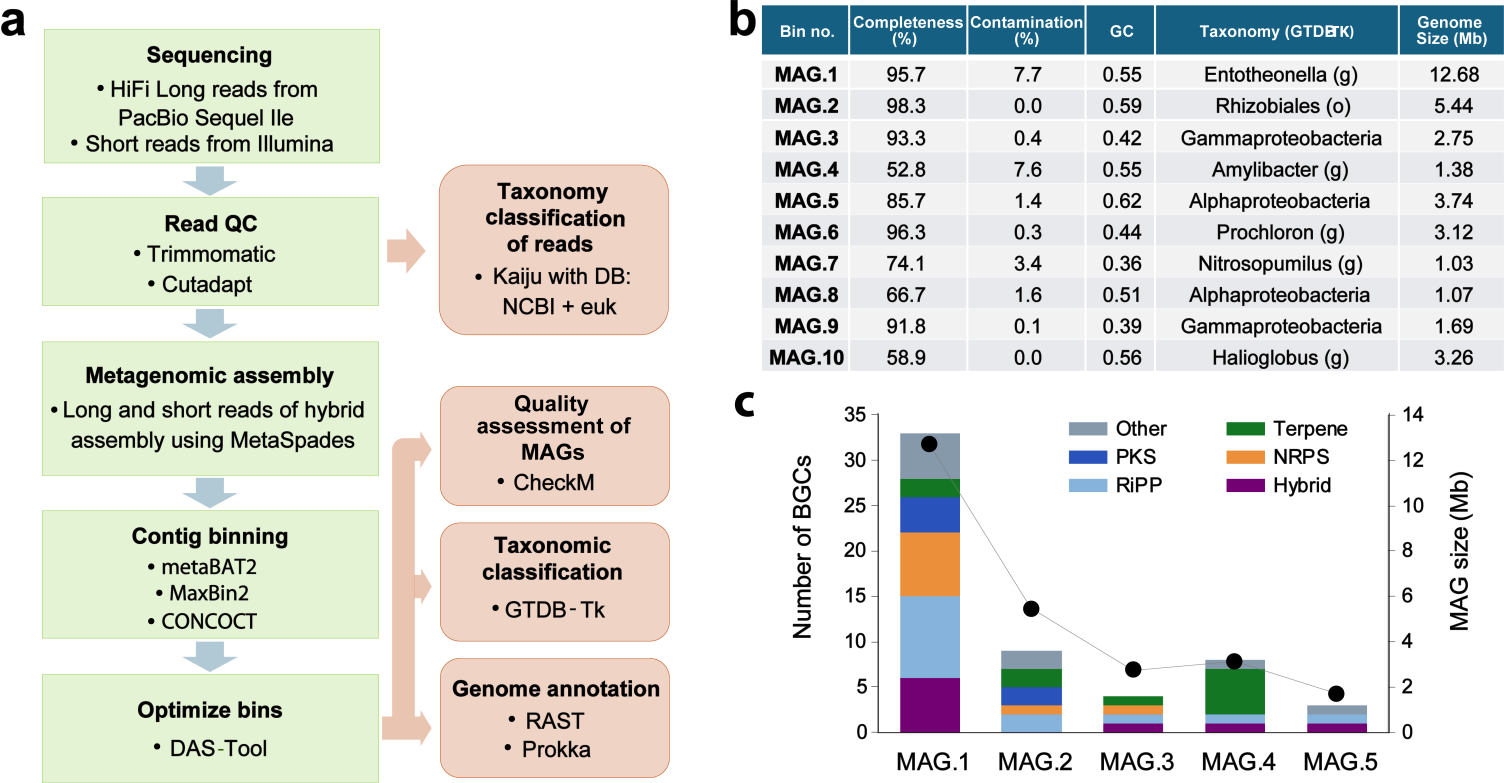
Genome-resolved metagenomics of the marine sponge *H. dokdoensis*. (a) The data processing workflow for the hybrid assembly and binning of *H. dokdoensis* metagenome sequencing data. The quality of MAGs was assessed using CheckM. MAGs with over 50% completeness and less than 10% contamination were selected for further analysis. (b) MAGs resolved from the *H. dokdoensis* metagenome. The taxonomic classification of each MAG was performed using GTDB-Tk. The MAG.1 with the largest genome size represents the genome of *Ca*. Entotheonella symbiont. (c) BGC abundance analysis of high-quality MAGs. BGC abundance was evaluated by the number of BGCs identified from each MAG using the antiSMASH pipeline. BGCs were color-coded according to their classification. Of the five MAGs, *Ca*. Entotheonella showed the highest number of BGCs (33 BGCs in total).

The peptide natural products halicylindramides have been previously isolated from the sponge *H. dokdoensis* (7). The high abundance of *Ca*. Entotheonella species in this sponge suggests that this bacterial symbiont could be the halicyclindramide producer. To assess this possibility, we performed genome-resolved metagenomics of *H. dokdoensis* to retrieve the genomes of its microbial symbionts. To this end, we employed an integrated approach that utilizes both short-read (Illumina Miseq) and long-read (PacBio Sequel IIe) sequencing for the *de novo* assembly of metagenome-assembled genomes (MAGs) (Fig. 3a). Taxonomic classification of metagenomic sequencing reads using Kaiju (31) revealed a microbial composition consistent with that obtained from 16S rDNA amplicon sequencing data (Fig. S3). The hybrid assembly of short (21.2 Gb) and long (24.8 Gb) sequencing reads generated a total of 37,614 contigs (>500 bp), cumulatively spanning 73.7 Mb in size (Table S2), which were resolved into putative genomes using a combination of different binning algorithms. This resulted in 10 high-quality MAGs (MAG.1–10) with >50% completeness and <10% contamination (Fig. 3b). Of these, 5 MAGs were nearly complete with >90% completeness (Table S3).

Each MAG was taxonomically classified using the Genome Taxonomy Database Toolkit (GTDB-Tk) (32). Of the 10 MAGs resolved, MAG.1, with a coverage of 533 and 95.7% completeness, was classified into the genus *Ca*. Entotheonella with an estimated genome size of 12.7 Mb (Table S3). To the best of our knowledge, this represents the largest and most complete *Ca*. Entotheonella MAG to date, surpassing the sizes and completeness of previously reported *Ca*. Entotheonella MAGs (GeneBank accession no. AZHW01, PPXO01, and AZHX01, summarized in Table S4). In addition, we also retrieved four additional high-quality MAGs (MAG.2, 3, 6, and 9) with >90% completeness and <1% contamination. The sizes of these MAGs were relatively small compared with that of MAG.1 (Table S3). The second-largest MAG (MAG.2; 5.4 Mb) was taxonomically affiliated with the class Alphaproteobacteria. The other three MAGs, each below 5 Mb in size, were taxonomically classified into the phylum Cyanobacteria (MAG.6; 3.1 Mb) and Gammaproteobacteria (MAG.3; 2.8 Mb and MAG.9; 1.7 Mb). Previous reports have indicated that genome size is proportionally correlated with the number of BGCs (33, 34). Therefore, we next investigated BGC abundance in the five high-quality MAGs with over 90% completeness using antiSMASH (antibiotics and secondary metabolite analysis shell) (Fig. 3c) (35). As anticipated, MAG.1, with the largest genome (12.7 Mb), contained the highest number of BGCs (33 BGCs), followed by MAG.2 (5 Mb; 9 BGCs) and MAG.6 (3 Mb: 8 BGCs). Fewer than five BGCs were found in MAG.3 and MAG.9 having smaller genomes (<3 Mb). These results align with those of the previous reports, indicating a strong correlation between genome size and BGC abundance (33), suggesting that the *Ca*. Entotheonella symbiont is likely to be the major contributor to the chemical diversity in *H. dokdoensis*.

### Genomic analysis of halichnodria sponge-associated Entotheonella

**Fig 4:**
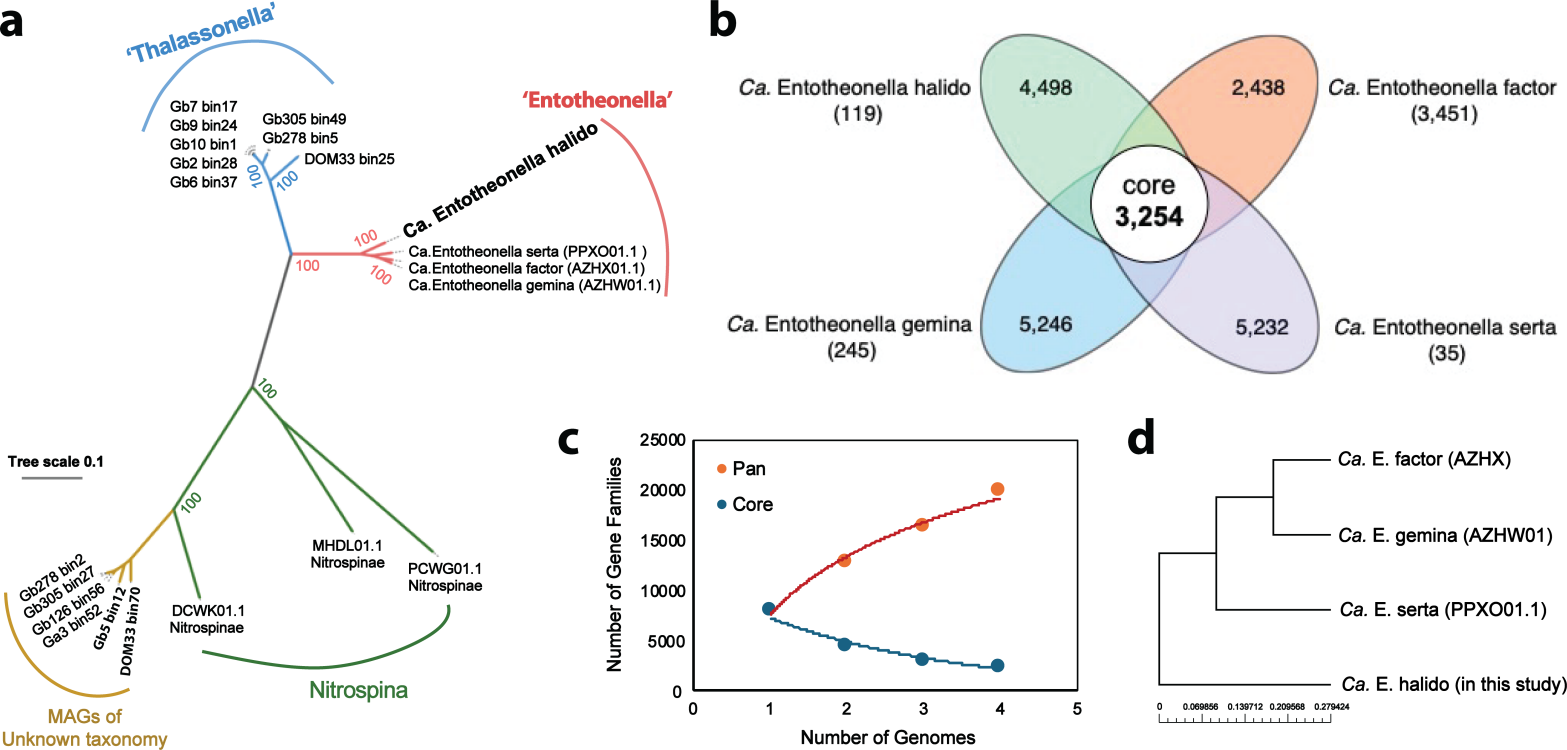
Comparative genomics of sponge-associated *Ca*. Entothenoella MAGs. (a) Phylogenomic analysis of *Ca*. Entotheonella halido and related Tectomicrobia MAGs. Three reference draft genomes from *Ca*. Entotheonella species (*Ca*. E. factor, *Ca*. E. gemina, and *Ca*. E. serta) and three draft genomes from the adjacent bacterial phylum Nitrospinae (MHDL01, PCWG01, and DCWK01) were included to infer the phylogenetic position of *Ca*. E. halido. (b) Flower diagram showing the core, accessory, and unique genes in the sponge-associated *Ca*. Entotheonella pangenome. Numbers in parentheses represent the number of unique genes in each MAG. (c) Core/pan-genome plot calculated over 50 iterations using BPGA analysis tool. (d) A phylogenetic tree is inferred from the alignment of core genes. *H. dokdoensis*-associated *Ca*. E. halido has evolved independently from the other three *Ca*. Entotheonella species associated with *T. swinhoei*.

The uncultivated candidate genus “*Entotheonella*” has been known to form symbiotic associations with marine sponges (28, 36). Recently, the Piel group reported the presence of two distinct *Ca*. Entotheonella phylotypes (37). Group I *Entotheonella* are highly host-specific and metabolically versatile, whereas group II *Entotheonella* is likely to be host-promiscuous symbionts or non-symbiotic contaminants from seawater. To date, only two sponge hosts have been identified to harbor *Ca*. Entotheonella symbionts, all belonging to group I, including *Theonella swinhoei* (26, 30) and *Discodermia calyx* (16, 38). *Halichondria dokdoensis* represents a new symbiotic host of *Ca*. Entotheonella species, which we named *Ca*. E. halido after its host name (Fig. 4a). *Ca*. E. halido is highly host-specific and rich in BGCs, and thus, we evaluated its taxonomic position in the *Entotheonella* phylogeny (Fig. 4a; Fig. S4). The inferred neighbor-joining phylogenetic tree revealed a distant evolutionary relationship of *Ca*. E. halido from both groups I and II members and formed an outgroup, cladding with *Ca*. E. palauensis. This probably suggests that *Ca*. E. halido and *Ca*. E. palauensis might together represent a new phylotype, although more members are needed to support this claim. The *Ca*. E. halido genome represents the largest *Ca*. Entotheonella genome (12.7 Mb) reported to date, surpassing the sizes of the previously reported Entotheonella genomes, including *Ca*. E. factor (9.7 Mb), *Ca*. E. serta (8.9 Mb), and *Ca*. E. gemina (9.9 Mb). To gain insight into the genetic diversity of sponge-associated *Ca*. Entotheonella symbionts, we performed a pan-genome analysis of the four available *Ca*. Entotheonella MAGs using the BPGA (bacterial pang-genome analysis) pipeline (39). The *Ca*. Entotheonella pan-genome is comprised of 34,280 genes in total, including 13,016 core (3,254 each), 17,414 accessory, and 3,850 unique genes (Fig. 4b). The core-pan plot indicates that the Entotheonella pan-genome is open. Thus, the size of the pan-genome will be expanded if more *Ca*. Entotheonella genomes are sequenced and added, whereas the size of the core genome remains relatively constant (Fig. 4c). The phylogeny inferred using the core genes suggested that *H. dokdoensis* sponge-associated *Ca*. E. halido has evolved independently from the other three *Ca*. Entotheonella species, all associated with the sponge *T. swinhoei* (Fig. 4d). A gene classification using the Kyoto Encyclopedia of Genes and Genomes (KEGG) database revealed a high sequence divergence in genes encoding two-component systems: 32 core, 71 accessory, and 103 unique genes (Fig. S5). It has been previously reported that the bacterial second messenger cyclic diguanylate determines host specialization, and its levels are regulated by two-component systems (40). This strongly suggests that two-component systems have also played a crucial role in *Ca*. Entotheonella’s adaptation to different symbiotic host environments.

### Biosynthetic potential of halichondria sponge-associated *ca*. Entotheonella

**Fig 5:**
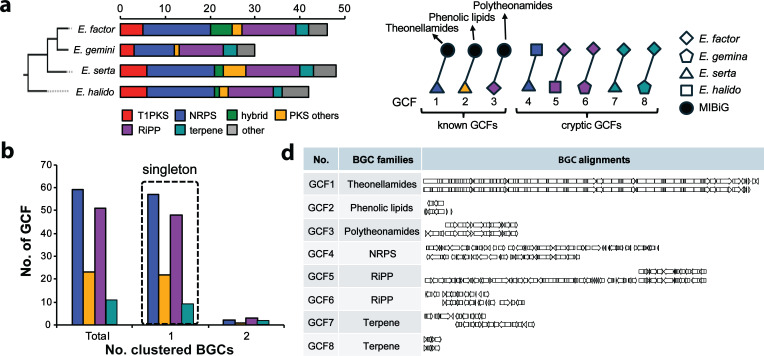
BGC diversity analysis of marine sponge-associated *Ca*. Entotheonella MAGs. (a) Comparison of BGC abundance and distribution across various BGC families. BGC abundance represents the total number of BGCs in each MAG, which might be overestimated in low-quality MAGs due to the possibility of duplicated counts of fragmented BGCs. BGCs identified through antiSMASH analysis were reclassified based on our criteria after manual inspection. (b) Prevalence analysis of GCFs across four sponge-associated Entothenoella MAGs. The prevalence of singleton GCFs was highlighted in a square box. No GCF was present with more than two BGCs. (c) Known GCFs and cryptic doubleton GCFs with the origin of each BGC distinguished by node shape. GCF1-3 are singletons of known BGCs, including BGCs of theonellamides, phenolic lipids, and polytheonamides. (d) Genotype comparison of BGCs clustered within the same GCF. BGCs in GCF1-3 were aligned with known BGCs from the MIBiG database, and GCF4-8 are doubletons, containing two BGCs originated from different MAGs.

Sponge-associated *Ca*. Entotheonella symbionts are recognized as a biosynthetically talented bacterial genus; however, no study has evaluated their biosynthetic diversity at a pangenome level. To gain insight into the biosynthetic diversity within the sponge-associated *Ca*. Entotheonella pan-genome, we conducted genetic similarity network analysis of BGCs using antiSMASH (35) and Big-SCAPE (41). The Big-SCAPE algorithm generates a BGC similarity network based on clustering using three metrics: the Jaccard index, an adjacency index, and domain sequence similarity. A total of 166 BGCs were identified in the *Ca*. Entotheonella pan-genome. Although the *Ca*. E. halido MAG is comprised of 257 contigs, other MAGs are highly fragmented with 962 contigs for *Ca*. E. factor, 3,146 contigs for *Ca*. E. serta, and 1,914 contigs for *Ca*. E. gemina. Since most BGCs are located at contig edges in these MAGs, the total of 166 BGCs is likely to be an overestimation. Manual examination of BGC annotations suggested that the sponge-associated *Ca*. Entotheonella symbionts are likely to contain 30–40 BGCs per MAG on average. This BGC abundance is comparable with that of *Streptomyces*, a genus renowned for having the highest number of BGCs. The most abundant BGC families in the *Ca*. Entotheonella genomes were NRPS (33%, 14 BGCs per MAG) and RiPP (27%, 11 BGCs per MAG) BGCs, followed by T1 PKS (5 BGCs per MAG) (Fig. 5a). The distribution of BGC families was highly similar among the *Ca*. Entotheonella MAGs. Compared with the *Streptomyces* genomes, which are rich in both T1PKS and NRPS BGCs, the *Ca*. Entotheonella MAGs are particularly rich in NRPS and RiPP BGCs, both of which produce peptide natural products. Notably, most of the *Ca*. Entotheonella NRPS BGCs contain only a single module (Fig. S6). This probably suggests that module duplication events are likely to be rare in “*Ca*. Entotheonella” genome evolution.

Next, the BGC diversity present in the *Ca*. Entotheonella pangenome was evaluated through genetic similarity network analysis that clusters BGCs into gene cluster families (GCFs). BGC clustering with two different cutoff values (0.3 and 0.7) produced the same results, classifying a total of 157 BGCs into 153 GCFs (Fig. 5b). Of these, 143 GCFs were singletons (no clustering), and five GCFs were doubletons (clustering of two BGCs). No GCF was identified with more than two BGCs. This result indicates that there is no common BGC present in all *Ca*. Entotheonella genomes, demonstrating a substantial BGC diversity present in the *Ca*. Entotheonella pangenome. The five doubleton GCFs (GCFs 4–8) were all cryptic, including one NRPS, two RiPPs, and two terpene BGCs (Fig. 5c and d). Only three singleton GCFs (GCFs 1–3) were clustered with known BGCs from the MiBIG (Minimum Information about a Biosynthetic Gene Cluster) database (42), which include BGCs of theonellamides (GCF1), phenolic lipids (GCF2), and polytheonamides (GCF3). These BGCs have been originally identified from the MAGs of *Ca*. E. serta (GCFs 1 and 2) (26, 43) and *Ca*. E. factor (GCF3) (27) and therefore uniquely present in these genomes. The result of BGC clustering indicates that the majority of BGCs are present in the *Ca*. Entotheonella pangenome possess unique genotypes that are distinct from each other and also from previously characterized known BGCs of other bacterial genera and thus have the potential to produce novel natural products. The distinct set of BGCs found in each *Ca*. Entotheonella MAG likely indicates that most of these BGCs were acquired and evolved after the establishment of symbiotic relationships with their marine sponge hosts.

### Biosynthesis of halicylindramides

**Fig 6:**
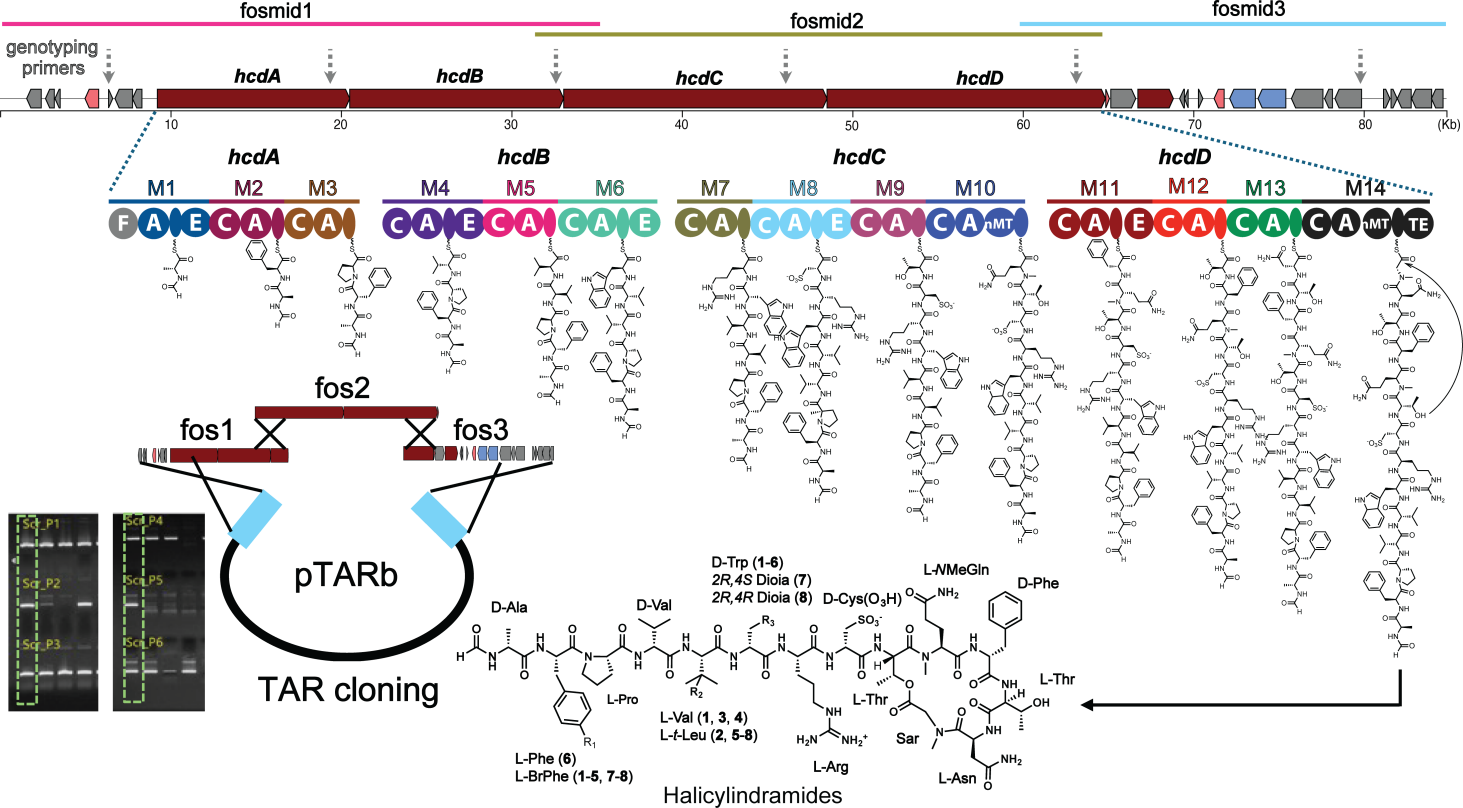
Proposed biosynthesis of halicylindramides through analysis of recovered BGC. A nearly complete halicylindramide (*hcd*) BGC was identified from the *H. dokdoensis* MAG. to recover the entire *hcd* BGC and facilitate subsequent cloning, a fosmid library was constructed using metagenomic DNA of *H. dokdoensis*. Illumina sequencing of three overlapping fosmids enabled the recovery of the full-length *hcd* BGC. this BGC was then captured into a BAC vector through TAR assembly of overlapping fosmids in yeast, and the successful assembly was confirmed by PCR-genotyping using six primer sets with amplification sites indicated by gray dotted arrows.

Halicylindramides (*hcd*) are peptide natural products isolated from the sponge *H. dokdoensis*. The highest biosynthetic potential observed in *Ca*. E. halido among other associated symbionts suggests that this symbiont would be the true halicyclindramide producer. We manually inspected the module organization of the nine NRPS BGCs identified in the *Ca*. E. halido MAG. Of these, one NRPS BGC (BGC 1.1) was identified to have a module organization mostly corresponding to the amino acid composition of the halincylindramides, based on antiSMASH adenylation domain specificity prediction and the presence of tailoring domains. However, the first NRPS gene was truncated, missing the modules for the first three amino acids (Ala-Phe-Pro). To recover the complete *hcd* BGC, we constructed the metagenomic DNA library of *H. dokdoensis* using a fosmid vector. This library was expanded to 20,000 unique clones to ensure sufficient coverage for recovering the full-length *hcd* BGC. Then, we screened the fosmid library using three PCR primer sets that bind across the partial *hcd* BGC, which led to the recovery of three overlapping fosmid clones (fosmids 1–3 in Fig. 6). Then, these three fosmid clones were sequenced using Illumina MiSeq. These fosmids contained the four full-length NRPS genes (*hcdA-D*), which together harbor 14 NRPS modules with substrate predictions corresponding to the amino acid sequence of the halicylindramides. This indicated that we successfully recovered the complete *hcd* BGC (~90 Kb) across three overlapping fosmids. The halicylindramides contain the first amino acid Ala formylated, and the previous result demonstrated that the amino acid formylation is catalyzed by the formylation domain present in the corresponding NRPS module (44). The detailed analysis of the first NRPS module by BLAST identified an additional formylation domain that was not predicted by antiSMASH. The presence of this rare formylation domain further verified that this NRPS BGC is indeed the halicylindramide BGC.

Since the sponge-associated *Ca*. Entotheonella symbiont has been unculturable (25), we next attempted to express the *hcd* BGC in genetically tractable heterologous hosts. The recovered three fosmid clones were assembled and captured into a shuttle BAC vector (*E.coli:yeast:Streptomyces*) in yeast using TAR (transformation-associated recombination) (45). The BAC-*Hcd* construct was transferred to the three well-known *Streptomyces* hosts: *Streptomyces coelicolor*, *Streptomyces albus,* and *Streptomyces roseosporus*. However, the HPLC and LC-MS analyses of the organic culture extracts failed to identify any BGC-specific metabolites including halicylindramides. Due to the possibility that the *Ca*. Entothenoella promoters are not recognized by *Streptomyces* sigma factors, we refactored the *hcd* BGC by exchanging three native promoters with well-characterized *Streptomyces* synthetic promoters (Fig. S7), but the promoter-engineered *hcd* BGC still failed to produce any halicylindramide-related metabolites in the *Streptomyces* hosts. This suggests that a more comprehensive refactoring, including codon optimization and cofactor supply, would be required to achieve the heterologous expression of secondary metabolites produced by the unculturable *Ca*. Entotheonella symbionts.

### Conclusion

Korean marine sponges have been a prolific source of bioactive natural products; however, their biosynthetic potential at the genetic level remains largely unexplored. Since sponge-associated microbial symbionts have been identified to be the original producers of many sponge-derived natural products, we conducted a metagenomic analysis of five different native marine sponges, all collected from the same coastal region of the East Sea of Korea to gain insight into their microbial communities and biosynthetic potential. The taxonomic analysis demonstrated that each sponge harbors a unique microbial composition, distinct from one another and from the surrounding seawater. Notably, we identified a unique association between Halichondria sponges and *Ca*. Entheonella bacteria, previously known to be metabolically rich, but had only been linked to the sponges *Theonella swinhoei* and *Discodemia calyx*. Thus, this finding expands the diversity of *Ca*. Entotheonella-associated symbiotic hosts. The sponge *H. dokdoensis* was unprecedently rich in the *Ca*. Entotheonella symbiont, comprising nearly 69% of its microbial community. This *Ca*. Entotheonella symbiont was also found to be phylogenetically divergent from the previously known other *Ca*. Entotheonella species, thus designated as *Ca*. E. halido. Through genome-resolved metagenomics, we successfully recovered the *Ca*. E. halido MAG, which represents the highest-quality and largest *Ca*. Entotheonella genome to date, consisting of 257 scaffolds with 533× coverage and a total size of 12 Mb.

Pan-genome analysis of the four sequenced *Ca*. Entotheonella MAGs revealed significant genetic diversity, with the highest diversity observed in genes encoding two-component systems. This suggests that two-component systems have likely played a crucial role in the adaptation of *Ca*. Entotheonella to distinct host environments. BGC network analysis also revealed remarkably high BGC diversity within the *Ca*. Entotheonella pangenome, with almost no overlapping BGCs between the MAGs. This suggests that the sponge-associated *Ca*. Entothenoella symbionts have acquired most of their BGCs after establishing their symbiotic association with sponge hosts. The Piel group previously identified two distinct phylotypes for sponge-associated *Ca*. Entotheonella: host-specific members (group I) and generalist colonizers (group II) (37). All previously characterized BGC-rich *Ca*. Entotheonella symbionts fall into the group I phylotype. However, *Ca*. E. halido was distant from both Groups I and II members, forming a separate outgroup in the phylogenetic tree. This likely suggests that sponge-associated *Ca*. Entotheonella symbionts do not form a strong phylogenetic lineage but have evolved independently by actively acquiring BGCs from surrounding bacteria as part of their specific symbiotic relationships with sponge hosts. This increasing number of BGCs may offer them an advantage by enhancing the defensive role of their hosts. We anticipate that the identification of additional *Ca*. Entotheonella symbionts from diverse sponge hosts will further expand both the phylogenetic and biosynthetic diversity of this bacterial genus.

BGC analysis also identified *Ca*. E. halido as the producer of the sponge-derived natural product halicylindramides. Since sponge-associated *Ca*. Entotheonella symbionts are yet unculturable, we cloned the *hcd* BGC from the *H. dokdoensis* metagenomic library and attempted to express it in heterologous hosts. Unfortunately, this BGC failed to produce halicylindramides in the *Streptomyces* hosts. Given that the majority of *Ca*. Entothenoella BGCs are cryptic, we are currently working on the development of a heterologous expression platform using synthetic biology approaches to facilitate the discovery of novel natural products from these metabolically talented sponge-associated *Ca*. Entotheonella symbionts.

## MATERIALS AND METHODS

### Sponge collection

Sponge samples were collected via scuba diving at depths of 10–25 m off the shore of Chuksan, East Sea of Korea (36°30′42.82″N, 129°27′8.46″E) from 2018 to 2023. Sponges were frozen immediately after collection, stored separately, and transported in an ice box with dry ice. The sponge samples were kept at −20°C until used. Sponge specimens were identified by analyzing their external appearances under microscope such as bone structures, sizes, and spicule shapes. The identification process was enhanced by linking molecular data to morphological traits. All the sponge specimens were deposited at the National Marine Biodiversity Institute of Korea (MABIK, South Korea) with the following voucher numbers: *Halichondria dokdoensis* (MABIK IV00173701), *Cliona celata* (MABIK IV00173703), *Halichondria* sp. (MABIK IV00173715), and *Mycale* sp. (MABIK IV00173713). In addition, 40 liters of seawater were collected and filtered through a 0.22 µm pore membrane (Merck Millipore) under vacuum. The solid residue was stored in RNAlater™ until DNA extraction.

### Metagenome sequencing

The frozen sponges were freeze-dried overnight, and the dried tissues were ground into fine powder using a mortar and pestle. DNA was then extracted from the resulting powder (250–500 mg) using the Qiagen DNeasy Powersoil extraction kit (QIAGEN) according to the manufacturer’s instruction. The surrounding seawater DNA was also extracted from the stored sample using the same method as for the sponge samples. Full-length 16S rDNA was amplified from the metagenomic DNA samples using universal primers (16S_27F and 16S_1492R in Table S6) and sequenced on the PacBio HiFi platform. The metagenomic DNA samples were also directly sequenced using Illumina MiSeq. Shotgun libraries were generated using NEBNext dsDNA Fragmentase (NEB), and sequencing was performed using the Miseq kit v.2 (Illumina). To improve the sequencing quality, we also sequenced a fosmid metagenomic DNA library using the long-read PacBio HiFi platform. The method for fosmid library construction is described in the cloning section below. A HiFi SMRTbell library (10 µL) was prepared using the PacBio SMRTbell Express Template Prep Kit 2.0, annealed with the Sequel II Bind Kit 2.2 and Internal Control Kit 1.0, and sequenced using the Sequel II Sequencing Kit 2.0 and Sequel II SMRT Cell 8M Tray (PacBio).

### Taxonomic profiling and phylogenetic analysis

The 16S rDNA amplicon sequencing reads from the PacBio Sequel IIe platform were analyzed using CLC Genomics Workbench Microbial Genomics Module (v 11.0) with predefined workflows for data quality control and taxonomic profiling (CLC Bio). Sequences were classified through operational taxonomic unit (OTU) clustering using the SILVA reference library with a 97% similarity threshold (20). For the metagenome sequencing data, taxonomic annotation of Illumina short reads was performed using Kaiju (v1.10) (31) with default settings. The 16S rDNA sequence of *Ca*. E. halido was extracted and analyzed using PhyloFlash v3.4 (46) with the SILVA rRNA gene database v138.1 (20). Additionally, a random selection of tectomicrobial 16S rDNA sequences, primarily selected from BGC-proliferative *Ca*. Entotheonella 16S sequences from previous studies (37), was included for phylogenetic analysis. These sequences were aligned using MUSCLE (47) and manually curated to improve accuracy. A phylogenetic tree was inferred using the Neighbor-Joining method with the Jukes-Cantor substitution model, using 500 bootstrap replicates for statistical support, all implemented in MEGA11 (48). The resulting phylogeny was visualized using the Interactive Tree of Life (iTOL v6) (49).

### Metagenome assembly and contig binning

Illumina or PacBio sequencing reads were processed by removing low-quality reads, sequencer adapters, and PCC1 vector sequences using Trimmomatic (v0.33) (50) and Cutadapt (v4.8) (51). Short and long reads were assembled together using metaSPAdes assembler (v3.15.5) (52). The assembled contigs were then binned using the MetaWRAP (v1.3) (53) pipeline with default parameters, employing the MaxBin2 (v2.2.7) (54), MetaBAT2 (v2.17) (55), and CONCOCT (v1.1) (56) binning tools. To enhance the binning quality, the binning results were refined via MetaWRAP’s bin refinement module (DAS_Tool v1.1) (57), applying a minimum completeness threshold of 50% and a maximum contamination limit of 10%. The completeness and contamination of the resulting genome bins were evaluated by CheckM (v1.0.2)’s lineage-specific workflow (58). The obtained bins were taxonomically classiﬁed using GTDB-Tk, v2.3.2 (32), implemented as an application in KBase (59). Bowtie2 (v2.5) (60) was used to align sequencing reads to each MAG, and the resulting alignment files were then converted, sorted, and indexed using SAMtools (v1.19) (61). The coverage of MAGs was determined using CoverM (https://github.com/wwood/CoverM) from the alignment data.

### Whole genome phylogenomic analysis

For the taxonomic classification of MAGs, dereplicated bins were created using the multiple sequence alignment generated using the GTDB-Tool Kit v2.4.0 (GTDB-Tk) alignment workflow (32) with FastTree (v2.1.11) (62), following default parameters. The resulting phylogenetic tree was then visualized and annotated using the Interactive Tree of Life (iTOL v6). This classification incorporated three reference MAGs of the “*Ca*. Entotheonella” species: “*Ca*. E. factor” (NCBI ID AZHW01), “*Ca*. E. gemina” (AZHX01), and “*Ca*. E. serta” (PPXO01). Additionally, three closely related draft genomes from the phylum Nitrospinae (MHDL01, PCWG01, and DCWK01) were included to provide a comparative framework.

### Genetic and BGC diversity analysis of MAGs

Pangenome analysis was conducted using the Bacterial Pan Genome Analysis (BPGA) tool v1.3 (39). Genome annotation was performed using Prokka (v1.14.6) (63). Orthologous genes were identified using USEARCH (v11) (64) with a similarity threshold of 0.5. No significant difference was observed in the result when the threshold value was changed to 0.3, 0.4, 0.6, or 0.7. Core- and pan-genome plots were generated by 500 iterative calculations. The functional annotation was carried out using the KEGG database (65) integrated within BPGA. For the BGC diversity analysis, BGCs were initially identified from the annotated MAGs using the genome mining pipeline antiSMASH v.7.1.0 (35). Each BGC classification generated by antiSMASH was manually inspected and re-classified according to our criteria. Genetic similarity network analysis of identified BGCs was performed using BiG-SCAPE (41) with a default similarity score cutoff (c = 0.3; threshold of 30%). The resulting sequence similarity matrices were then visualized using Cytoscape (v.3.10.2) (66).

### Cloning of halicyclindramide BGC

A fosmid library with 8X coverage was constructed using the metagenomic DNA of the sponge *H. dokdoensis* and the CopyControl Fosmid Library Production Kit (BIOSEARCH TECHNOLOGIES) according to the manufacturer’s protocol. The library was PCR-screened using six primer sets (Table S6) that target across the *hcd* BGC. Whole-cell PCR screening was performed using MyTaq polymerase (Bioline), and the positive hists were end-sequenced by Sanger sequencing. The overlapping fosmids were assembled using Transformation-Associated Recombination (TAR) in yeast, following the previously published protocol (45). Approximately 500 bp sequences homologous to the distal flanking regions of the *hcd* BGC were PCR-amplified from each fosmid and cloned into the pTARb shuttle BAC vector, creating a cluster-specific capture vector. Fosmid assembly was performed by co-transforming 200 ng of the HpaI-linearized capture vector and 300 ng of each fosmid, linearized with PsiI, XbaI, or SbfI, into yeast using a spheroplast yeast transformation (67). Transformants were selected on His-dropout SC agar, and a total of 48 colonies were picked and cultured in the same dropout liquid media for DNA mini-prep. Plasmids were extracted from 2 mL of the overnight culture using the Zymolyase lysis protocol (ZYMO RESEARCH). Miniprep DNAs were transformed into *E. coli* EC100 for long-term storage. The successful cloning of the complete *hcd* BGC was confirmed by Illumina sequencing (Macrogen).

## Data Availability

All raw and processed data in this study are available under NCBI BioProject ID PRJNA1116994 and accession numbers for all data items are provided in the Table S5. The 16S rRNA gene sequence of *Ca*. E. halido is available under the accession number PQ114579.

## AFTER EPUB

*[This article was published on 22 November 2024 with an error in the Acknowledgments. A missing contribution was corrected in the current version, posted on 6 December 2024.]*

## SUPPLEMENTAL MATERIAL

Fig. S1 spectrum.02355-24-s0001.tif Taxonomic profiles of microbial communities in Korean marine sponges.

Fig. S2 spectrum.02355-24-s0002.tif Microscopic observation of the sponge *Halichondria dokdoensis*.

Fig. S3 spectrum.02355-24-s0003.tif Taxonomic profile of *H. dokdoensis* metagenome based on Kaiju (DB: NCBI+euk) analysis.

Fig. S4 spectrum.02355-24-s0004.tif Phylogenetic tree of *Ca*. Entotheonella to infer the taxonomic position of *Ca.* E. halido.

Fig. S5 spectrum.02355-24-s0005.tif KEGG pathway analysis of *Ca*. Entotheonella pangenome.

Fig. S6 spectrum.02355-24-s0006.tif Genotypes of NRPS BGCs identified from the *Ca*. E. halido MAG (MAG.1).

Fig. S7 spectrum.02355-24-s0007.tif Promoter engineering of the hcd BGC.

Supplemental material spectrum.02355-24-s0008.docx Supplementary figure legends.

Supplemental data spectrum.02355-24-s0009.xlsx Taxonomic assignments for all operational taxonomic units.

Supplemental tables spectrum.02355-24-s0010.docx Tables S1 to S6.

ASM does not own the copyrights to Supplemental Material that may be linked to, or accessed through, an article. The authors have granted ASM a non-exclusive, world-wide license to publish the Supplemental Material files. Please contact the corresponding author directly for reuse.
