# Supplementary figures and images for: Metagenomic insights and biosynthetic potential of Candidatus Entotheonella symbiont associated with Halichondria marine sponges
Source: Microbiol Spectr. 2024 Nov 22;13(1):e02355-24. doi: 10.1128/spectrum.02355-24 (PMC11705928; doi:10.1128/spectrum.02355-24)

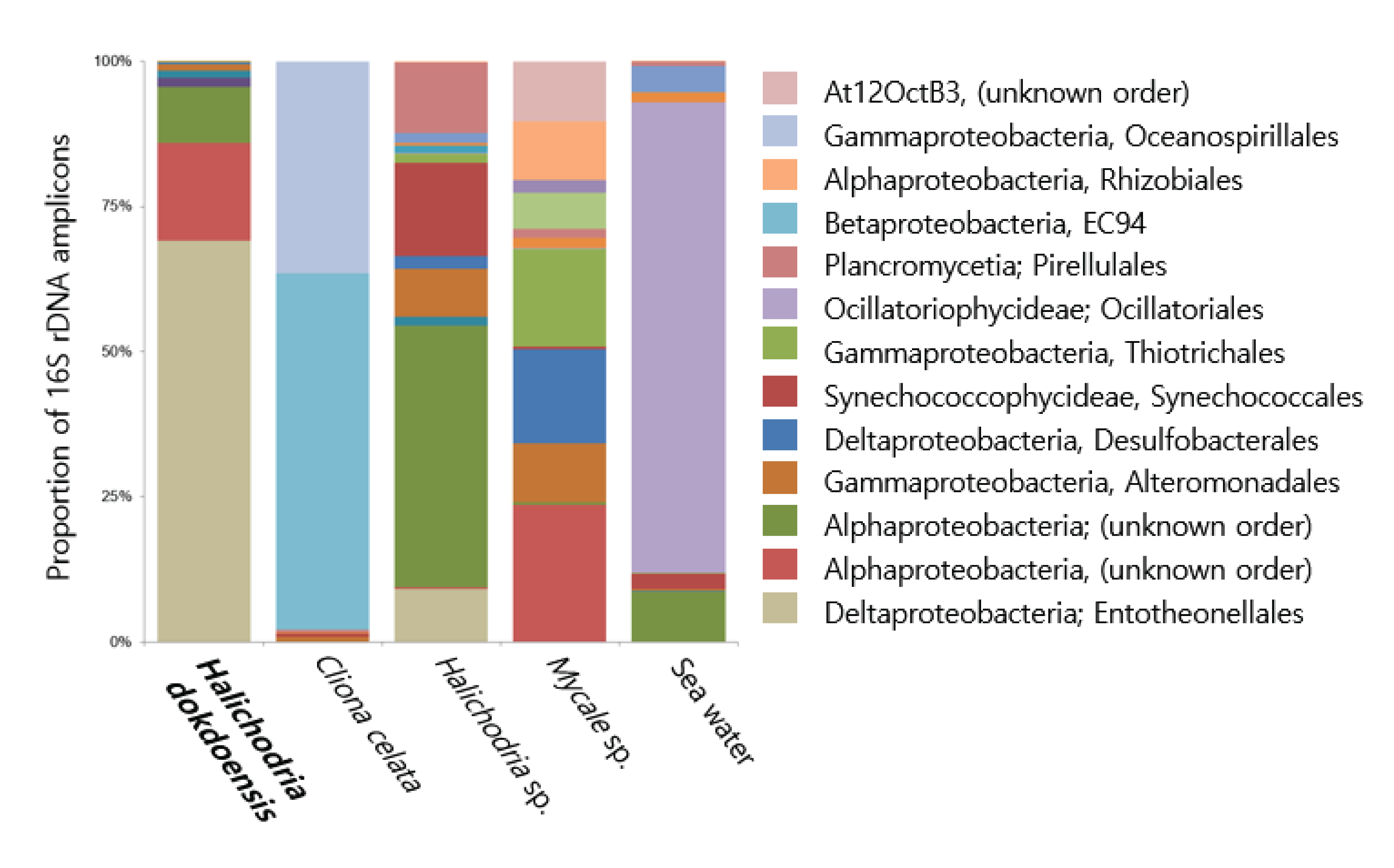

Supplement: Fig. S1 — Taxonomic profiles of microbial communities in Korean marine sponges. [file spectrum.02355-24-s0001.tif]

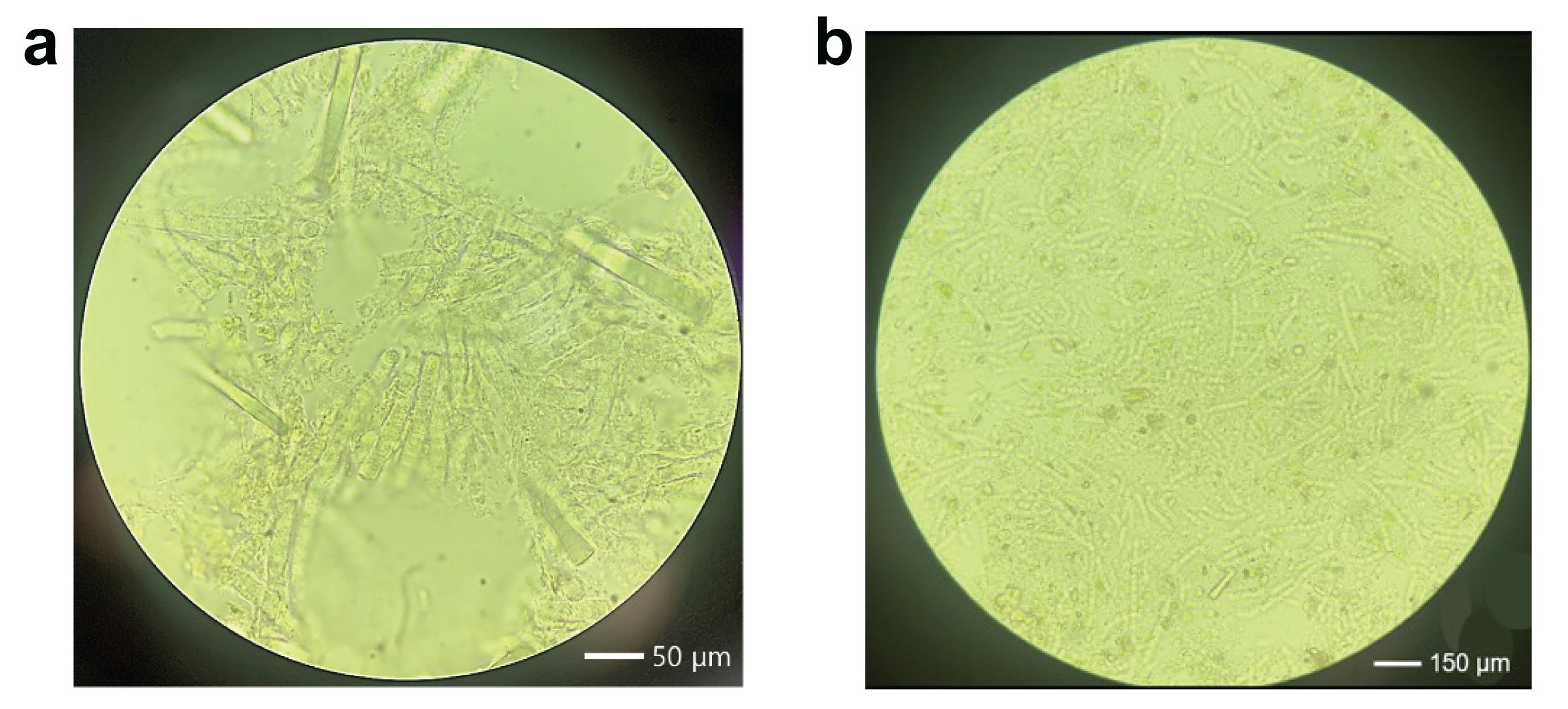

Supplement: Fig. S2 — Microscopic observation of the sponge Halichondria dokdoensis. [file spectrum.02355-24-s0002.tif]

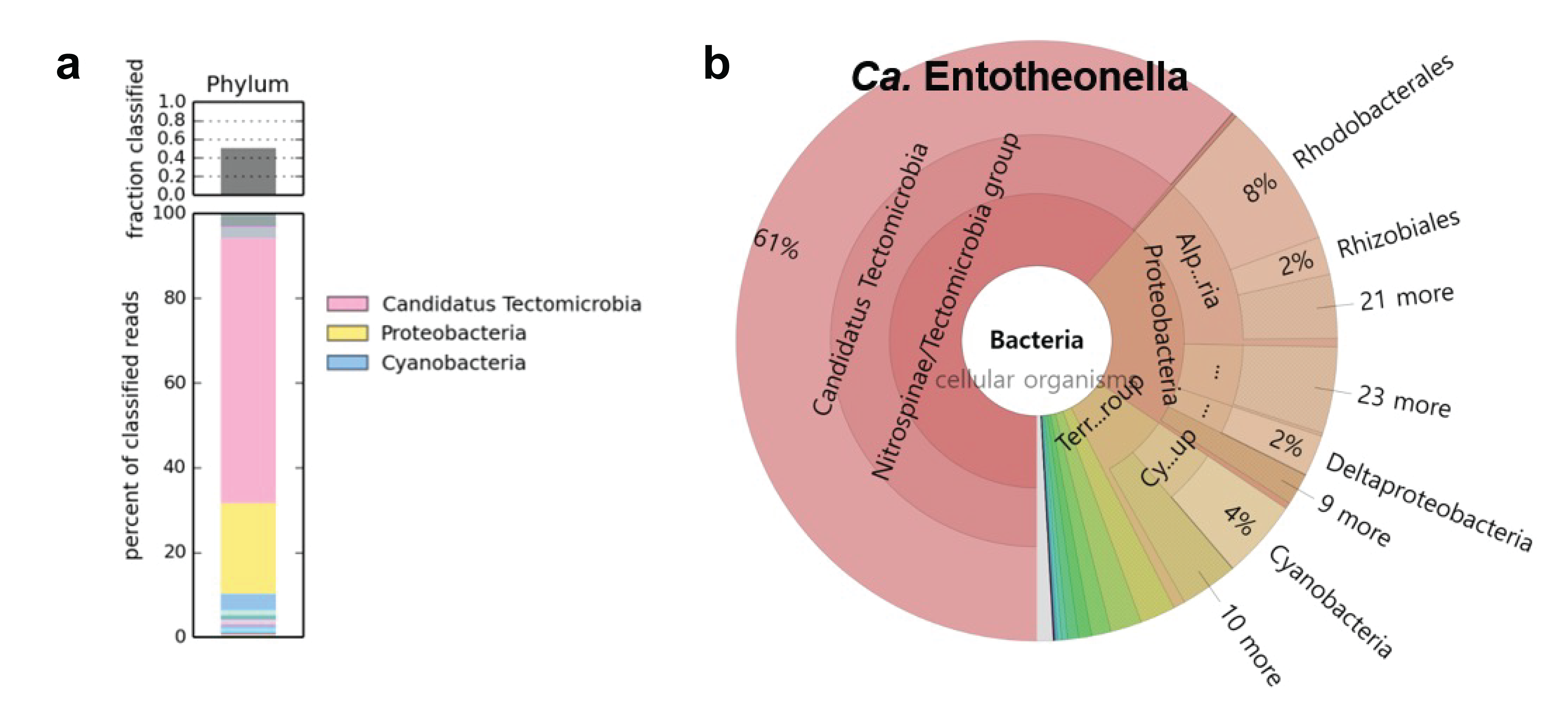

Supplement: Fig. S3 — Taxonomic profile of H. dokdoensis metagenome based on Kaiju (DB: NCBI+euk) analysis. [file spectrum.02355-24-s0003.tif]

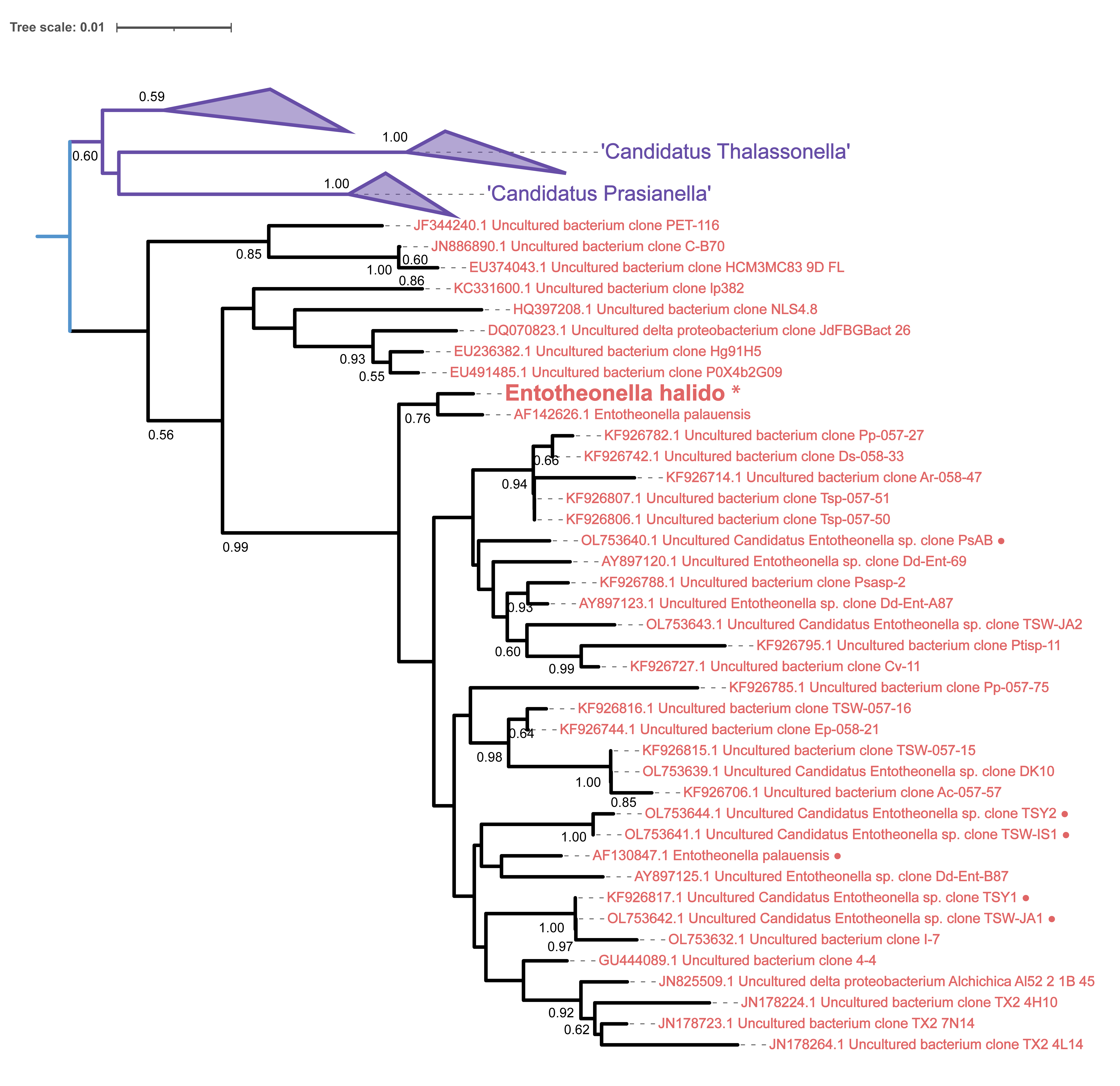

Supplement: Fig. S4 — Phylogenetic tree of Ca. Entotheonella to infer the taxonomic position of Ca. E. halido. [file spectrum.02355-24-s0004.tif]

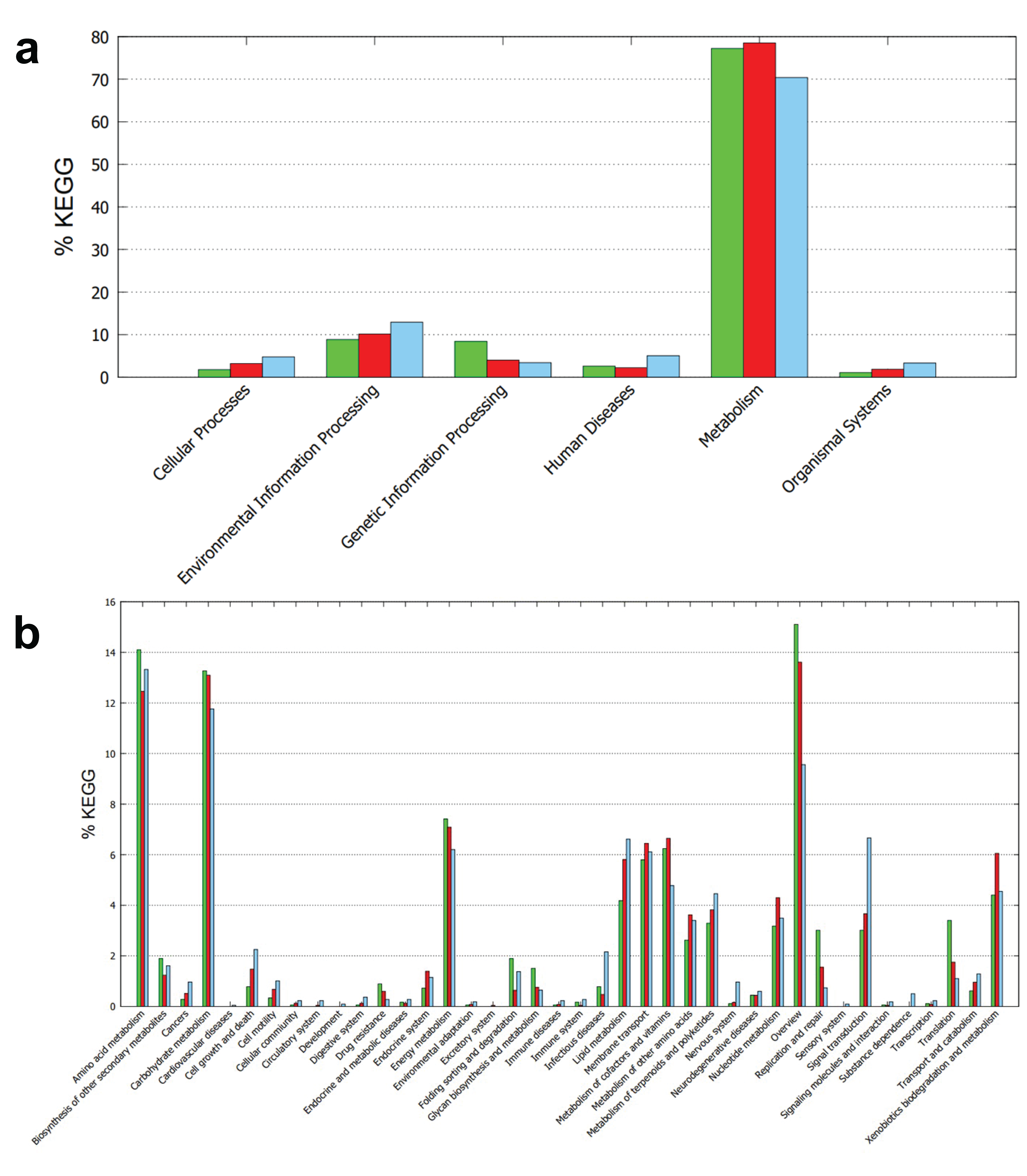

Supplement: Fig. S5 — KEGG pathway analysis of Ca. Entotheonella pangenome. [file spectrum.02355-24-s0005.tif]

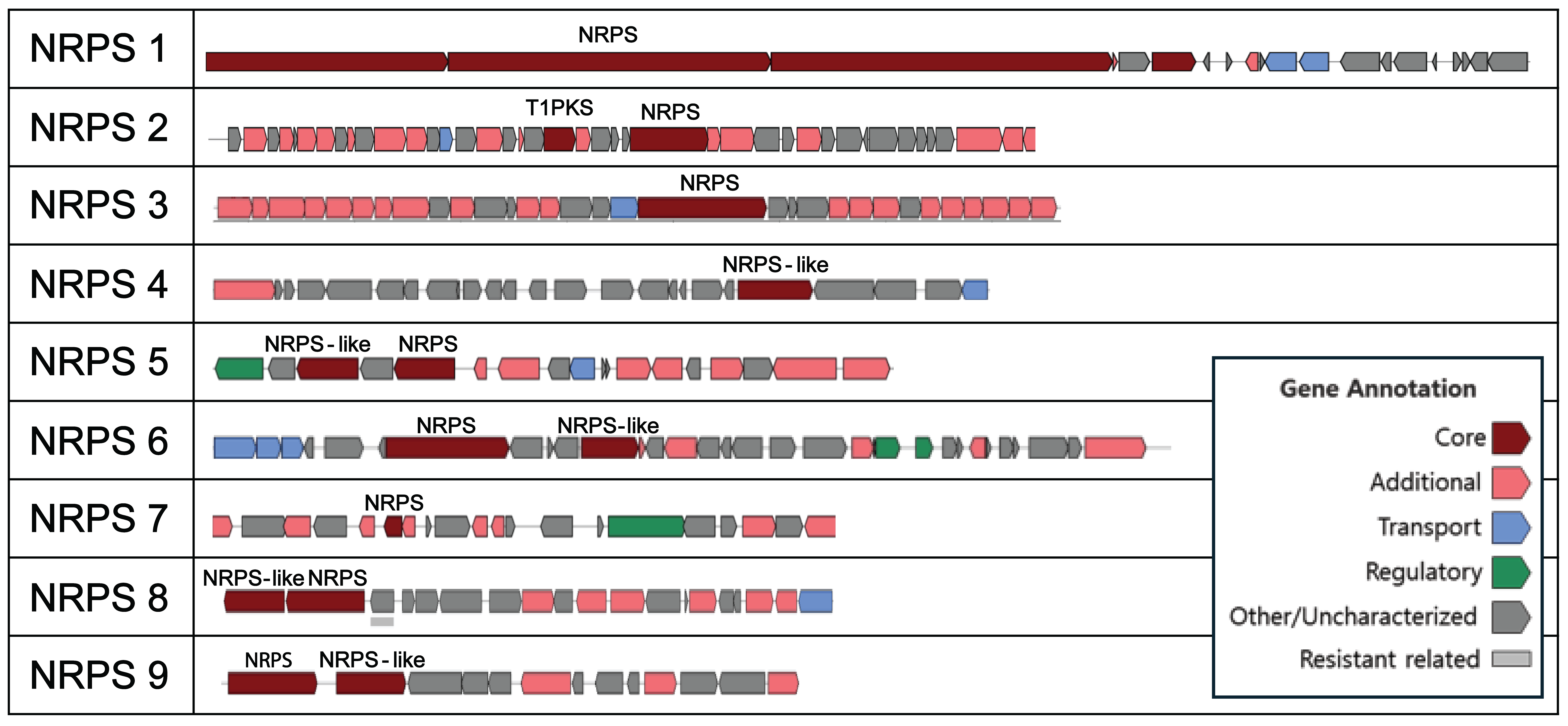

Supplement: Fig. S6 — Genotypes of NRPS BGCs identified from the Ca. E. halido MAG (MAG.1). [file spectrum.02355-24-s0006.tif]

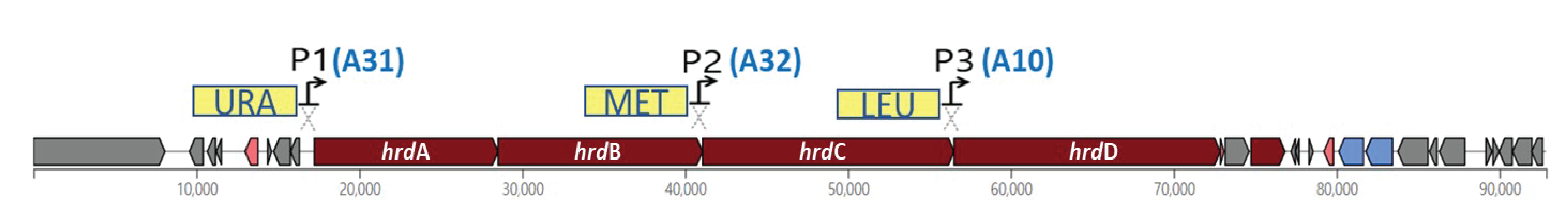

Supplement: Fig. S7 — Promoter engineering of the hcd BGC. [file spectrum.02355-24-s0007.tif]
