# Supplementary material for: Metagenomic insights and biosynthetic potential of Candidatus Entotheonella symbiont associated with Halichondria marine sponges
Source: Microbiol Spectr. 2024 Nov 22;13(1):e02355-24. doi: 10.1128/spectrum.02355-24 (PMC11705928; doi:10.1128/spectrum.02355-24)
Supplement: Supplemental material — Supplementary figure legends. [file spectrum.02355-24-s0008.docx]

**SUPPLEMENTARY FIGURE LEGENDS**

**Fig. S1. Taxonomic profiles of microbial communities in Korean marine sponges.** Relative abundances of microbial phyla are calculated from 16s rDNA amplicon sequencing data. Only taxa with abundance > 1% are displayed.

**Fig. S2. Microscopic observation of the sponge *Halichondria dokdoensis*. a** A thin section of the sponge sample was frozen, sliced, and then observed under an optical microscope. The microscopic image reveals a dense population of filamentous microorganisms along with sponge spicules. **b** The microscopic image depicts the sediment observed under a microscope after the sponge was blended and centrifuged at 500g. After discarding supernatant, the remaining sediment predominantly contained filamentous *Entotheonella*.

**Fig. S3. Taxonomic profile of *H. dokdoensis* metagenome based on Kaiju (DB: NCBI+euk) Analysis.** **a** Taxonomic classification of metagenomic reads at the phylum level. The top bar chart illustrates the fraction of classified reads as determined by Kaiju. The bottom chart shows the relative abundance of bacterial phyla within the sample. The most abundant phylum is *Ca.* Tectomicrobia (pink), followed by Proteobacteria (yellow) and Cyanobacteria (blue). **b** Detailed Taxonomic Breakdown of Bacterial Communities. The Krona Plot provides a detailed hierarchical breakdown of the bacterial community structure, visualizing the taxonomic distribution from the phylum to the order level. The innermost ring represents the phylum level, with *Ca.* Tectomicrobia comprising 61% of the total bacterial reads. The subsequent rings display more detailed classifications, such as the Nitropsinae/Tectomicrobia group, Proteobacteria, and Cyanobacteria. The outermost segments highlight specific orders within these phyla, including Rhodobacterales, Rhizobiales, and Deltaproteobacteria, among others. The percentages indicate the relative abundance of each group within the bacterial community.

**Fig. S4. Phylogenetic tree of ‘*Ca.* Entotheonella’ to infer the taxonomic position of *Ca.* E. halido.** Bootstrap values greater than 50% are indicated. Entotheonella bacteria previously associated with the production of bioactive natural products are marked with a red dot. The scale bar represents 0.01 nucleotide substitutions per site.

**Fig. S5. KEGG pathway analysis of Ca. Entotheonella pangenome. a** Overview of major KEGG pathway categories. **b** Detailed KEGG pathway analysis. The bar graphs were color-coded according to their classifications as core, accessroy and unique genes. The signal transduction category includes genes encoding two-component systems that are highly diverse in the *Ca.* Entotheonella pangenome.

**Fig. S6. Genotypes of NRPS BGCs identified from the *Ca*. E. halido MAG (MAG.1).** These BGCs were classified as NRPS BGCs based on the antiSMASH analysis result. A total of 9 NRPS BGCs were identified within the *Ca*. E. halido MAG, of which only one NPRS BGC (NRPS 1) contained more than one module. The NPRS1 corresponds to the halicylindarmide BGC.

**Fig. S7. Promoter engineering of the hcd BGC**. The strong Streptomyces synthetic promoters, designated as A31, A32 and A10, were inserted immediately upstream of the core NRPS biosynthetic genes *hcdA*, *hcdB*, and *hcdC*. Each promoter cassette contains a unique yeast auxotrophic marker to facilitate the selection of positive colonies: *URA3-A31*, *MET15-A32*, and *LEU2-A10*. Promoter engineering was performed using CRISPR/Cas9-aided TAR in yeast.
