# Supplementary material for: Metagenomic insights and biosynthetic potential of Candidatus Entotheonella symbiont associated with Halichondria marine sponges
Source: Microbiol Spectr. 2024 Nov 22;13(1):e02355-24. doi: 10.1128/spectrum.02355-24 (PMC11705928; doi:10.1128/spectrum.02355-24)
Supplement: Supplemental tables — Tables S1 to S6. [file spectrum.02355-24-s0010.docx]

**SUPPLEMENTARY TABLES**

**Table S1. HiFi read statistics from 16s rDNA amplicon sequencing**


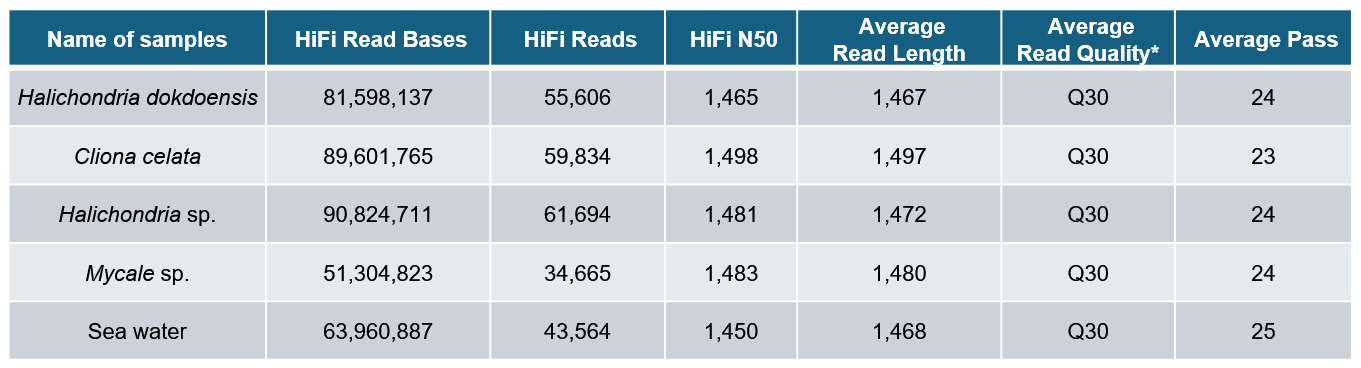


- HiFi Read Bases: Total bases of HiFi reads.
- HiFi Reads: The number of reads in the HiFi read.
- HiFi Read N50: 50% of all bases come from HiFi reads longer than this value.
- Average Read Length: The mean length of HiFi reads.
- Average Read Quality(Phred Quality Score): The mean quality of HiFi reads (A quality score of 30 to a base(Q3) means the chances of having base call error are 1 in 1000).
- Average Pass: The mean number of passes in the HiFi read.

**Table S2. Summary of sequencing and assembly statistics**


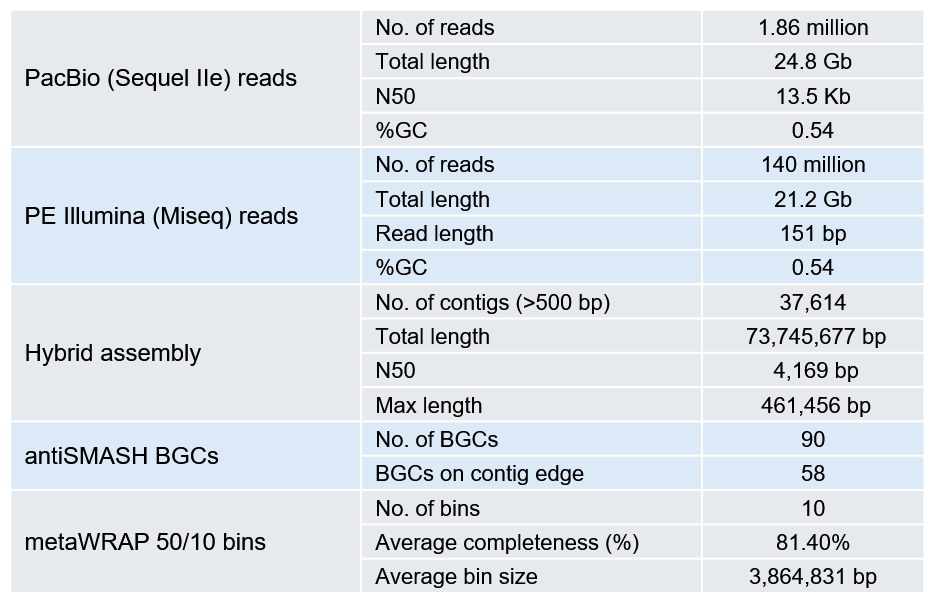


**Table S3. Genome information of recovered MAGs from the metagenome of sponge *H. dokdoensis***


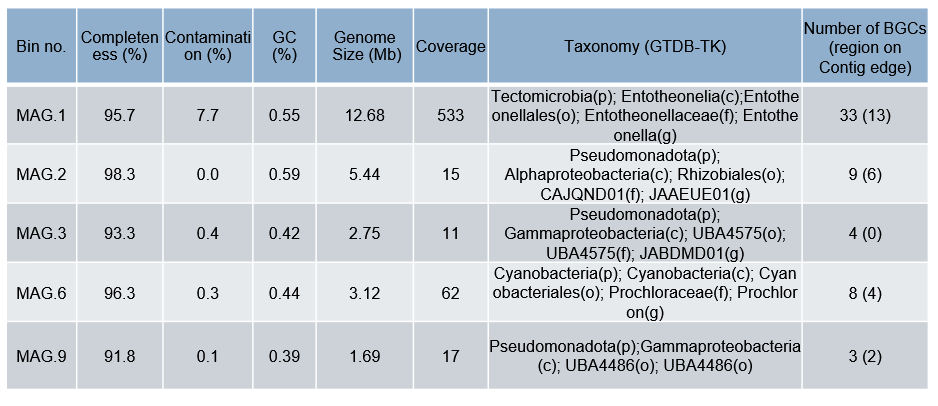


- The coverage of MAGS was calculated using CoverM (<https://github.com/wwood/CoverM>)
- The number of BGCs was detected by AntiSMASH.

**Table S4. Comparison of Genome Information of Entotheonella MAGs from NCBI and newly recovered Ca. E. halido MAG in this study**


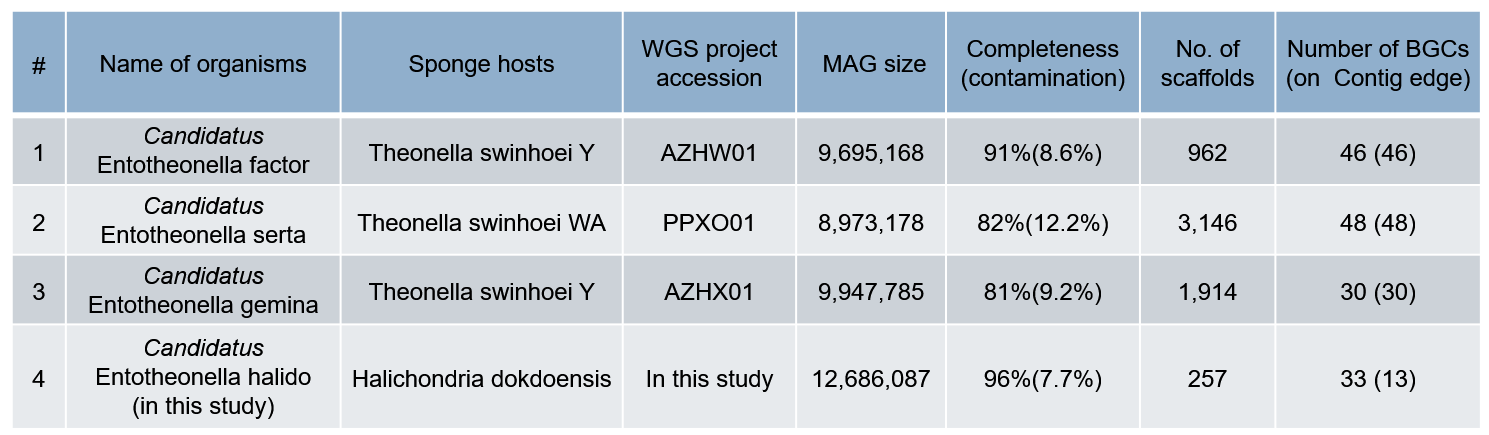


**Table S5. NCBI accessions under BioProject PRJNA1116994**

| Type of NCBI data | **NCBI Accession** | **Description** |
| --- | --- | --- |
| Biosample SAMN42797919 | SRR30038024 | Raw Illumina sequencing reads |
| Biosample SAMN42797919 | SRR30038023 | Raw PacBio sequencing reads |
| Biosample SAMN41554045 | JBFTEW000000000 | Scaffolds of MAG.1 (*Ca*. Entotheonella halido) |
| Genbank | PQ114579 | 16S rRNA gene sequence of MAG.1  (*Ca*. Entotheonella halido) |
| Biosample SAMN43580339 | SAMN43580339 | Raw PacBio HiFi reads of 16s rRNA amplicons from metagenome of sponge *Halichondria dokdoensis* |

**Table S6. Primers used in this study**

| No. | Oligo Name | 5` - Oligo Seq - 3` | Descriptions |
| --- | --- | --- | --- |
| 1 | pTARb-TRP-FW | TCTGACTGGGTTGGAAGGCA | pTARb end-sequencing primers |
| 2 | pTARb_MSC_RV | ATACTCAACTGCGTTACGCG | pTARb end-sequencing primers |
| 3 | fos1-1-aF | CAGTGACACGCTCATTGCCT | Screening fosmids |
| 4 | fos1-1-aR | CGAGCCAAAACACTCCGACA | Screening fosmids |
| 5 | fos1-3-aF | TTGCAAGTCGATCTGCCGTC | Screening fosmids |
| 6 | fos1-3-aR | GGATTGTCGGGGAGCAGTTG | Screening fosmids |
| 7 | fos23-aF | GACAAACGGCTCGTCGCTTA | Screening fosmids |
| 8 | fos23-aR | GCAAGTAGTCCACGTTCGGG | Screening fosmids |
| 9 | fos23-cF | GCAGTACGGGCGTCATTTCA | Screening fosmids |
| 10 | fos23-cR | GAGCTCGTAGTGTTTGCGCT | Screening fosmids |
| 11 | fos1-12-aF | CTGGAGGCAATCCTTCGCTG | Screening fosmids |
| 12 | fos1-12-aR | AAATCGTCGTGAACCAGCCC | Screening fosmids |
| 13 | fos1-23-aF | GTTGGTCCCAGCCAAAGTCC | Screening fosmids |
| 14 | fos1-23-aR | GACTCTAAATCCCGCCGCTG | Screening fosmids |
| 15 | 16S_27F | AGAGTTTGATCMTGGCTCAG | 16s rDNA amplicons |
| 16 | 16S_1492R: | TACGGYTACCTTGTTACGACTT | 16s rDNA amplicons |
